# *In Vitro* Transformation of Protopanaxadiol Saponins in Human Intestinal Flora and Its Effect on Intestinal Flora

**DOI:** 10.1155/2021/1735803

**Published:** 2021-10-22

**Authors:** Meiyu Zhang, Yizhu Wang, Yongxi Wu, Fangtong Li, Mingxin Han, Yulin Dai, Fei Zheng, Hao Yue

**Affiliations:** Jilin Ginseng Academy, Changchun University of Chinese Medicine, Changchun 130117, China

## Abstract

Protopanaxadiol (PPD)-type ginsenosides are the main ginsenosides in ginseng (*Panax ginseng* C. A. Meyer) with potential therapeutic effects on diseases related to intestinal flora imbalance. This study aimed to investigate the *in vitro* metabolism of protopanaxadiol ginsenosides in human intestinal flora and their effect on the flora. Rapid resolution liquid chromatography coupled with quadruple-time-of-flight mass spectrometry (RRLC-Q-TOF MS) was utilised for the transformation of ginsenoside constituents for sample identification. Using 16S rDNA gene sequencing technique, the effect of PPD-type ginsenosides on gut microflora was analysed based on the indices of microflora diversity and gut microflora. The sample was transformed for 6 h, and the metabolites were ginsenoside Rb1, Rc, Rb2, Rb3, CO, Gyp-IX, Gyp-XVII, CMc-1, F2, Rg3, CK, Rh2, and PPD. The metabolites were CK, Rh2, and PPD when the samples were transformed for 60 h. The intestinal microflora were subjected to high-throughput sequencing using the Illumina MiSeq 2500 sequencing platform. In comparison with the faecal sample from the blank group, the protopanaxadiol saponin group significantly increased the relative abundance of Firmicutes and significantly decreased Bacteroidetes and Proteobacteria at the phylum level, whereas it significantly increased the relative abundance of *Prevotella_9*, *Faecalibacterium*, and *Dialister* and significantly decreased *Escherichia-Shigella*, *Dorea*, and *Lachnoclostridium* at the genus level. This study provides a basis for the determination of the pharmacodynamic material basis and pharmacodynamic targets of PPD-type ginsenosides based on the intestinal flora.

## 1. Introduction

**Figure 1:**
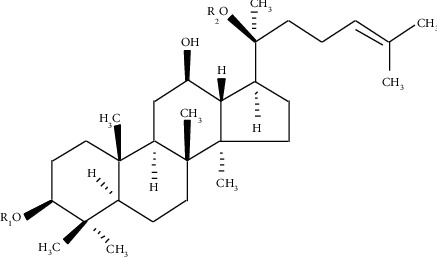
The chemical profile of PPD from *P. ginseng*.

Ginseng (*Panax ginseng* C. A. Meyer) is a precious oriental herb that has been used in traditional Chinese medicine for thousands of years, both as a disease-healing drug and a general tonic. *P. ginseng* is thought to be helpful in providing immunity against COVID-19 [1]. Many ginseng-based foods such as ginseng beverages, ginseng candy, ginseng tea, and ginseng honey tablets have been gradually developed worldwide [2]. According to the type of aglycones' structure, ginsenosides are divided into three types, including the oleanane, protopanaxadiol (PPD), and protopanaxatriol (PPT) types [3]. PPD-type ginsenosides (Figure 1 and Table 1) account for 45%–60% of the total ginsenosides [4]. They can inhibit oxidant stress, enhance immunity, lower blood sugar, resist tumour cells, and exhibit anti-inflammatory properties [5, 6].

After oral administration, most ginsenosides can interact with the intestinal flora in the intestines, thus transforming into secondary ginsenosides and being absorbed by the body [7]. PPT-type ginsenosides can be metabolised by the human intestinal flora, and the transformation products are mainly formed by the loss of sugar residues to form transformed products, while the secondary ginsenosides and aglycones are the material basis for the pharmacological effects of ginseng *in vivo* [8]. Moreover, the gut microbiome includes all the microbes living in the human gut, affecting human health and disease treatment. The balance of intestinal flora is closely related to the health and disease of the host. Adjusting the imbalance of intestinal flora, promoting probiotics, inhibiting pathogenic bacteria, correcting the imbalance of intestinal flora, and achieving a new balance are among the biological mechanisms of ginseng for the treatment of diseases [9, 10]. Previous research by this group showed that the extract of ginseng and the seed of Zizyphus jujuba var. spinosa changes the structure and diversity of gut microbiota in rats with spleen deficiency syndrome and balances the metabolic process [11].

In this study, the intestinal flora of healthy individuals was examined *in vitro*. Rapid resolution liquid chromatography-quadrupole-time-of-flight mass spectrometry (RRLC-Q-TOF MS) detection technology was used to fit the original ginseng glycol-type saponin group in the human intestinal flora in metabolic pathways. Then, we analysed the original-type ginseng diol saponin group after the structure of intestinal flora; this study aimed to reveal the original ginseng glycol-type saponin group in the law of metabolism in the body, the metabolic characteristics, and the effect of the PPD-type ginsenosides on the structure of intestinal flora.

## 2. Materials and Methods

### 2.1. Materials

Reference standards of ginsenosides Rb1, Rc, Rb2, Rb3, Rd, Gyp-IX, Gyp-XVII, CO, C-Mc1, F2, Rg3, CK, Rh2, and PPD were obtained from Shanghai Yuanye Biotechnology Co., Ltd. (Shanghai, China). Chromatographically pure acetonitrile was obtained from TEDIA Co., Ltd. (Fairfield, Ohio, USA). Dimethyl sulfoxide and n-butanol were obtained from Tianjin Damao Chemical Reagent Factory (Tianjin, China). General anaerobic medium (GAM) was obtained from Beijing Land Bridge Technology Co., Ltd. (Beijing, China). Chromatographically pure formic acid was obtained from Aladdin Co., Ltd. (Shanghai, China). Methanol and ethyl acetate were obtained from Tianjin Xintong Fine Chemical Co., Ltd. (Tianjin, China). The MinElute® PCR Purification Kit was obtained from Tiangen Co., Ltd. (Beijing, China). VnF and VnR were obtained from Shanghai Yingjun Biotechnology Co., Ltd. (Shanghai, China).

### 2.2. Preparation of Standard Solutions

Ginseng ginsenosides Rb1, Rc, Rb2, Rb3, Rd, Gyp-IX, Gyp-XVII, CO, C-Mc1, F2, Rg3, CK, Rh2, and PPD were accurately weighed and dissolved in a 10 mL volumetric flask with methanol solution at a constant volume and then shaken evenly to obtain a standard solution with concentrations of approximately 1 and 0.5 mg/mL.

### 2.3. Conditions for the RRLC-Q-TOF MS Analysis

Mass spectrometric parameters: different mobile phase compositions (methanol and water, methanol, and 0.1% formic acid in water, acetonitrile and water, acetonitrile, and 0.1% formic acid in water) and gradient elution procedures were investigated. A solvent system consisting of 0.1% formic acid in water (A) and acetonitrile (B) was selected as the mobile phase by gradient elution. A Zorbax Extend-C18 column (100 mm × 2.1 mm, 3.5 *μ*m) was used for gradient elution. The optimal elution conditions were as follows: 0–5 min, 0%–15% B; 5–10 min, 15%–19% B; 10–13 min, 19%–25% B; 13–15 min, 25%–28% B; 15–18 min, 28%–28% B; 18–22 min, 28%–30% B; 22–25 min, 30%–35% B; 25–30 min, 35%–40% B; 30–35 min, 40%–60% B; 35–38 min, 60%–80% B; 38–40 min, 80%–100% B; 40–42 min, 100%–100% B; column temperature, 35°C; flow rate, 0.5 mL/min; injection volume, 5 *μ*L. Mass spectrometric parameters: under the negative ion mode, the parameters of the electrospray ionisation mass spectrometry were optimised, and the internal parameters of the ion trap were optimised automatically based on the ionic strength of the ginsenosides. The electrospray ion source parameters, electrospray ion source, and electrospray negative ion mode were manually optimised. The mass scanning range was 100.0–1,200.0 m/z. The drying gas temperature was set to 350°C with a nebuliser pressure set of 255 kPa and a voltage of 3,500 V. The fragmentor voltage was set to 175 V. The taper hole voltage was set to 65 V.

### 2.4. Preparation of Isolated Intestinal Flora from Humans

Fresh faeces were provided by Chinese healthy male volunteers in the Affiliated Hospital of Changchun University of Chinese Medicine, Changchun, China. The experimental procedures were approved by the Medical Ethic Committee at the Affiliated Hospital of Changchun University of Chinese Medicine (Approval No. CCZYFYLL2014-049). Mixed fresh human faecal samples (10 g) were collected from 10 healthy male volunteers. These volunteers did not take any medicine for 3 months before the study. The samples were anaerobically incubated at 37°C for 24 h.

### 2.5. Preparation and Treatment of the Incubation System of Original Ginsenosides

Total ginsenosides were prepared according to the Chinese Pharmacopoeia (2020). The following parameters were used for the extraction and purification of PPD-type ginsenosides: macroreticular resin, D101; sample concentration, 15 mg/mL; absorption time, 12 h; and mobile phase, 75% ethyl alcohol. PPD-type ginsenosides including ginsenoside 19.23% Rb1, 17.26% Rc, 21.80% Rb2, 8.87% Rb3, and 23.77% Rd were obtained.

GAM and culture of the intestinal bacteria were added to the blank control group (K). In the experimental group (P), the gut microbiota solution of GAM and PPD-type ginsenosides were added. The sample was anaerobically incubated at 37°C and 100 rpm for 0, 6, and 48 h. The reaction mixtures were then extracted thrice with ethyl acetate for each extraction. The remaining residues were re-extracted thrice with n-butanol for each extraction. Then, the ethyl acetate and n-butanol layers were mixed homogeneously. Solvent recovery was conducted by decompression and then diluted to the desired volume with methanol. The solutions were filtered through a 0.22-*μ*m filter membrane before being injected for analysis.

### 2.6. Collection of Faecal Specimens

GAM and an appropriate amount of normal human gut microbiota liquid were added to the blank group (K). In the experimental group (P), the gut microbiota solution of GAM, human gut microbiota liquid, and PPD-type ginsenosides were added. Both groups were collected and placed in an anaerobic incubator for aseptic operation in an anaerobic environment. The samples were then cultured for 24 h under anaerobic conditions at 37°C and 100 rpm. The sample was centrifuged at 11,000 rpm for 5 min. The supernatant was discarded, and the precipitate was placed in liquid nitrogen for 30 min and stored in a refrigerator at −80°C.

### 2.7. Preparation of Faecal DNA for High-Throughput Sequencing Analysis

DNA was extracted using an MN NucleoSpin 96 Soil DNA kit, according to the manufacturer's instructions. The sample was treated and split; impurities were removed, inhibitors; DNA binding was conducted; the silicon matrix membrane was cleaned and dried; DNA was eluted; and the tube was transferred. The DNA was stored at −80°C until further processing. For the 16S rDNA using primers 27FAGG: (TTTGATYNTGGCTCAG) and 1492R: (TASGGHTACCTTGTTASGACTT), PCR reactions were performed in triplicate with 50 *μ*L of mixture containing 2.5 *μ*L of VnF, 2.5 *μ*L of Q5 high-fidelity DNA polymerase, 1.0 *μ*L of high GC Enhancer, 25 *μ*L of buffer, 10 *μ*L of dNTP, and 40–60 ng of template DNA. Initial denaturation was conducted at 95°C for 5 min, 95°C for 30 s, and 50°C for 30 s, with a final extension at 72°C for 7 min, followed by 30 cycles. Finally, all PCR products were quantified using ImageJ software and pooled together. High-throughput sequencing analysis of bacterial rRNA genes was performed on the purified and pooled samples using the Illumina HiSeq 2500 platform (PE250) at Biomarker Technologies Corporation, Beijing, China.

### 2.8. Bioinformatics and Statistical Analyses

USearch software was used to cluster tags at a 97% similarity level to obtain OTU, and taxonomic annotations were made for OTU based on Silva and Unite databases. The representative OTU sequences were compared with the microbial reference database to obtain the corresponding species' taxonomic information. Then, the community composition of each sample was counted at the phylum and genus levels, and the community structure of the sample at the phylum and genus taxonomic levels was plotted using QIIME software and R language tools.

## 3. Results and Discussion

### 3.1. Component Analysis of PPD Ginsenosides in the Intestinal Flora

RRLC-Q-TOF MS was performed to qualitatively analyse different ginsenosides during the biotransformation of PPD-type ginsenosides, and the obtained PPD-type ginsenosides and the precise molecular weight and retention time of the transformation products were obtained. Using tandem mass spectrometry (MS/MS), the characteristic fragment information of the parent ions was obtained through the rupture of the parent ions at the appropriate collision energy. Based on the above information and comparison with relevant literature, the identified components and their MS data are listed in Table 2.

**Figure 2:**
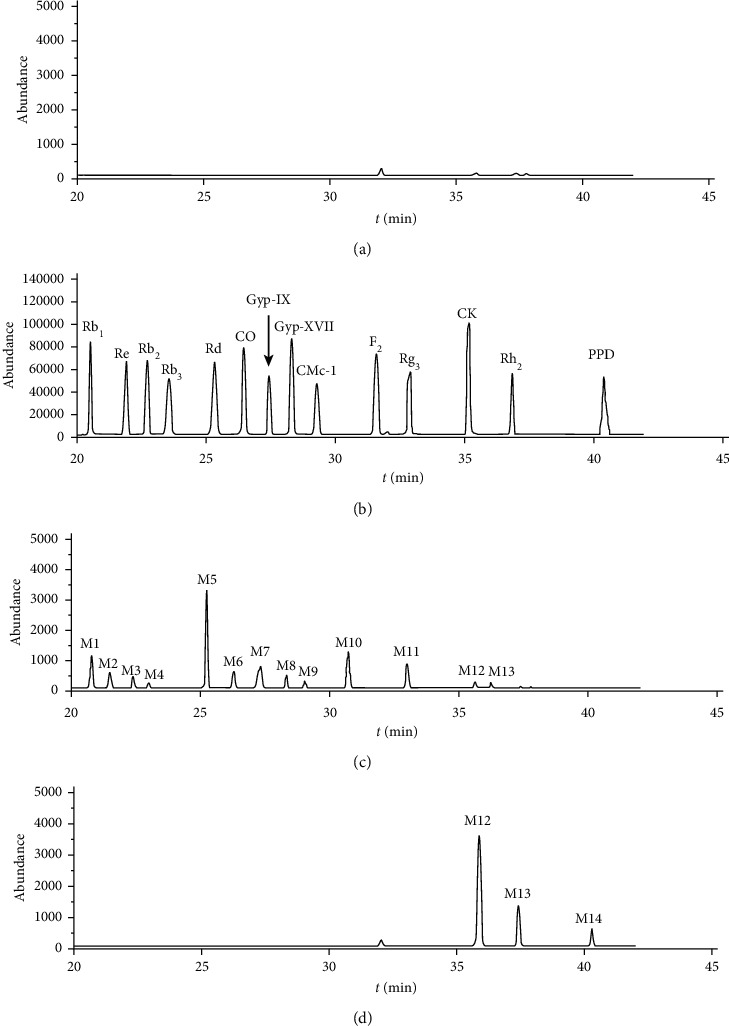
Total ion chromatogram of the transformation of ginsenosides in the PPD-type ginsenosides in the human intestinal flora in (a) blank group, (b) control group, (c) 6 h, and (d) 48 h.

**Figure 3:**
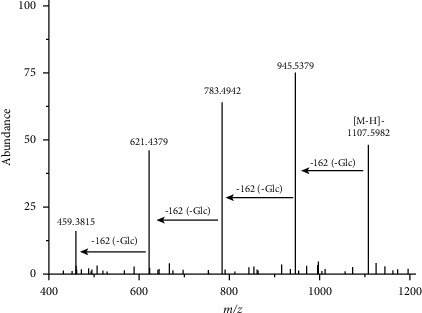
Tandem mass spectrometry of the metabolites of protoginsenoside Rb1 in the human intestinal flora.

The peak of M1 showed an [M-H]^−^ ion at m/z 1,107.5982, indicating that its molecular formula is C_54_H_92_O_23_, with a retention time of 20.217 min. As illustrated in Figure 2, in the MS/MS spectrum, the second-order mass spectrometry showed fragment ions at m/z 945.5379, 783.4942, 621.4379, and 459.3815. M1 resulted in an [M-H]^−^ ion at m/z 459.3815 via the successive elimination of four glucoses, and it can be identified as ginsenoside Rb1 (Figure 3). In combination with the results of mass spectrum analysis [12], M2, M3, and M4 were eluted at 20.924, 21.629, and 22.473 min, respectively, and their molecular formula was C_53_H_90_O_22_. Thus, M2, M3, and M4 were deduced to be ginsenosides Rc, Rb2, and Rb3, respectively. M6 is ginsenoside CO, M7 is gypenoside Gyp-IX, M8 is gypenoside Gyp-XVII, M9 is ginsenoside CMc-1, M11 is ginsenoside F2, M11 is ginsenoside Rg3, M12 is Compound K, M13 is ginsenoside Rh2, and M14 is ginsenoside PPD.

### 3.2. Metabolic Pathways of PPD-type Ginsenosides in Human Gut Flora

Ginsenoside Rb1 was converted into ginsenoside Rd by removing the C20 glucose. The glucose at the C3 position was transformed into gypenoside Gyp-XVII, and ginsenoside Rc was transformed into ginsenoside Rd at the C20 position of furan arabinose. Ginsenoside CMC-1 was obtained from glucose at C3, and ginsenoside Rb2 was obtained from pyran arabinose at C20 and transformed into ginsenoside Rd. Ginsenoside Rb3 could be converted into ginsenoside Rd from xylose at C20. Gypenoside Gyp-IX and ginsenoside Rd were converted into ginsenoside F2 by removing C3 glucose. Ginsenoside Rd can be converted into ginsenoside Rg3 by removing C20 glucose. Ginsenoside F2 and ginsenoside Rg3 can be converted into ginsenoside CK and ginsenoside Rh2 by removing C20 glucose , and ginsenoside PPD can be obtained by removing C20 and C3 glucose, respectively. Gypenoside Gyp-XVII removed C20 glucose, ginsenoside CMC-1 removed C20 furan arabinose, ginsenoside CO removed C20 pyran arabinose, and gypenoside Gyp-IX could remove C20 xylose and transform it into ginsenoside F2. Ginsenoside F2 can be converted into Compound K by removing C3 glucose and finally converted into ginsenoside PPD.

The results of accurate molecular mass and MS/MS tandem mass spectrometry data, identification of PPD-type ginsenosides metabolites, and fitting of metabolic pathways are as follows: ginsenoside Rb1 converts to seven metabolites, with the pathway of Rb1⟶Rd⟶F2⟶CK⟶PPD, Rb1⟶Gyp-XVII⟶F2⟶CK ⟶ PPD, Rb1⟶Rd⟶Rg3⟶Rh2⟶PPD; ginsenoside Rc converts to seven metabolites, with the pathway of Rc⟶Rd⟶F2⟶CK⟶PPD, Rc⟶Rd⟶Rg3 ⟶Rh2⟶PPD, Rc⟶C-Mc1⟶F2⟶CK⟶PPD; ginsenoside Rb2 converts to seven metabolites, with the pathway of Rb2⟶Rd⟶F2⟶CK⟶PPD, Rb2⟶Rd ⟶Rg3⟶Rh2⟶PPD, Rb2⟶CO⟶F2⟶CK⟶PPD; and ginsenoside Rb3 converts to seven metabolites, with the pathway of Rb3⟶Rd⟶F2⟶CK⟶PPD, Rb3⟶Rd ⟶Rg3⟶Rh2⟶PPD, Rb3⟶Gyp-IX⟶F2⟶CK ⟶PPD. After 6 h of sample transformation, the metabolites were Rb1, Rc, Rb2, Rb3, CO, Gyp-IX, Gyp-XVII, CMc-1, F2, Rg3, CK, Rh2, and PPD. When the sample was transformed for 60 h, the metabolites were CK, Rh2, and PPD.

### 3.3. Alpha Diversity Analysis

**Figure 4:**
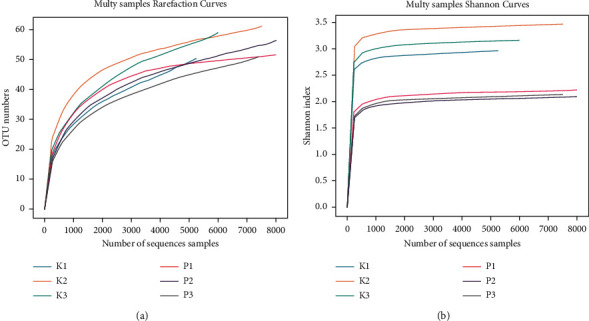
Richness and diversity of the gut microbiota. (a) Rarefaction curve. (b) Shannon curve.

Shannon and rarefaction analyses revealed that each sample was adequately sequenced and tended to be saturated, and the sequencing depth of the gut microbial environment was sufficiently captured in each sample and suitable for further analysis (Figure 4(a) and 4(b)).

### 3.4. Beta Diversity Analysis

**Figure 5:**
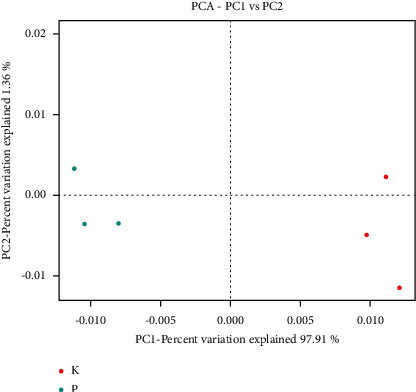
PCA of bacterial components in samples.

Beta diversity analysis compared the similarity of species diversity among different samples. Principal component analysis (PCA) can be used to classify multiple samples and further demonstrate species diversity differences among samples. The two ranking PCs, PC1 and PC2, described 97.91% and 1.36% of the total variability in the original observations, respectively. The PCA results showed that the structural composition of gut microbiota was different between groups K and P (Figure 5). The aggregation of samples suggested similarities within each sample within each group and independence between the two groups.

### 3.5. Key Phylotypes of Gut Microbiota

**Figure 6:**
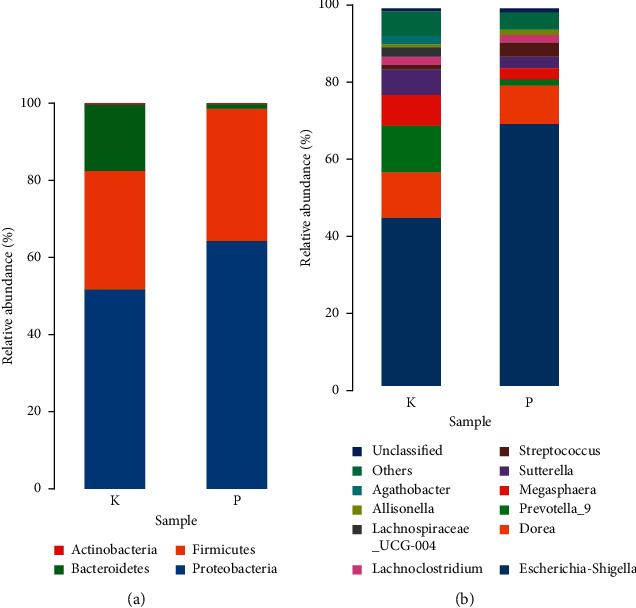
Structure of each group of samples: (a) phylum level and (b) genus level.

Figure 6(a) shows the differences in the species structure of intestinal flora between the two groups at the phylum level. The bacterial flora structure of the two groups was mainly composed of four dominant phyla: Firmicutes, Bacteroidetes, Proteobacteria, and Actinobacteria. This result is consistent with the research results of Valentina and Cecilia [13]. Approximately 30.51% of sequences (Figure 6(a)) in mice from the K group were classified as belonging to the phylum Firmicutes, whereas only 34.59% of sequences in mice from the P group were classified as belonging to this phylum. The relative abundance of Proteobacteria was 62.60% and 51.65% in mice from the P and K groups, respectively. The relative abundance of Bacteroidetes was 2.60% in mice from the P group and 16.64% in mice from the K group. Overall, the P group conspicuously changed the microbial composition in the model group with more Firmicutes and Proteobacteria but fewer Bacteroidetes. Wang et al. [14] found that after DSS administration, the proportion of Firmicutes was significantly decreased in the model group. In contrast, in the ginseng treatment group, the proportion of Firmicutes increased. This finding was consistent with our results. Ginseng polysaccharides have a prebiotic-like effect on OACS rats by simultaneously stimulating the growth of most important probiotics. *Bacteroides* spp. ginseng polysaccharides improved the intestinal metabolism and absorption of certain ginsenosides and reinstated the perturbed holistic gut microbiota [15]. This finding is consistent with our results. After ginsenoside administration, the abundance of Bacteroidetes was significantly reduced. Hence, ginsenoside also has a regulatory effect on the intestinal flora of normal people and can prevent diarrhoea caused by the imbalance of bacterial flora.

At the genus level (Figure 6(b)), 11 bacterial genera were isolated from the three groups at the genus level. Bacteria with high relative abundance include *Escherichia-Shigella*, *Sutterella*, *Streptococcus*, *Prevotella_9*, *Lachnoclostridium*, and *Megasphaera*. Compared with group K, the relative abundance of *Escherichia-Shigella* in group P was increased, whereas that of *Dorea*, *Prevotella_9*, and *Megasphaera* decreased. In the K group, approximately 44.68% of the sequences were classified as *Escherichia-Shigella*, and approximately 12.71% of the sequences were classified as *Preprevella_9*. *Escherichia-Shigella* had a high correlation with digestive tract symptoms. Hence, it also had a great influence on the “enteric-brain” axis. The treatment of mice with the probiotic bacterium *Lactobacillus rhamnosus* JB-1 reduces stress- and depression-like behaviour in a vagus nerve-dependent manner [16]. Administration of the original ginsenosides may ameliorate depression. *Prevotella* is the dominant bacteria in the intestinal tract, and Huws et al. [17] found that *Prevotella* is an inflammation-related bacterium that can cause diarrhoea. Therefore, the administration of proginsenediol ginsenosides can prevent and treat diarrhoea. Song et al. [18] reported the effect of ginseng on the intestinal flora of middle-aged obese Korean women, in which the relative abundance of *Dorea* was high in obese people but decreased after ginseng was administered, indicating that *Dorea* is related to human obesity. Ginseng has significant effects on the treatment of obesity, and current research results can provide a basis for clinical treatment.

**Figure 7:**
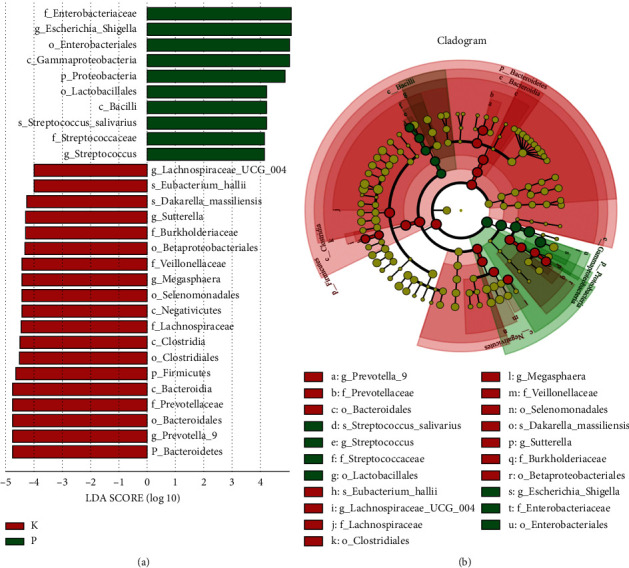
(a) Distribution of LDA value and (b) LEfSe analysis of evolutionary branch.

Linear discriminant analysis effect size (LEfSe) can determine the colony species with statistical differences among different groups. The histogram of LDA value distribution and the evolutionary branch diagram of LEfSe analysis showed that the LDA scores of the K and P groups were significantly higher than the present value of 4 (Figure 7(a) and 7(b)). A total of 31 species differed between the two groups. In the P group, *o_Lactobacillales*, *c_Bacilli*, *g_Streptococcus*, *s_Streptococcus_salivarius*, *f_Streptococcaceae*, *f_Enterobacteriaceae*, *g_Escherichia_Shigella*, *o_Enterobacteriales*, *c_Gammaproteobacteria,* and *p_Proteobacteria* were relatively abundant, and statistically significant differences were observed (*p* < 0.05). In the P group, *g_Lachnospiraceae_UCG_004*, *s_Eubacterium_hallii*, *s_Dakarella_massiliensis*, *g_Sutterella*, *f_Burkholderiaceae*, *o_Bacteroidales*, *o_Betaproteobacteriales*, *f_Veillonellaceae*, *g_Megasphaera*, *o_Selenomonadales*, *c_Negasphaera*, *c_Bacteroidia*, *f_Prevotellaceae*, *o_Bacteroidales*, *g_Prevotella_9*, *p_Firmicutes*, *o_Clostridiales*, *c_Clostridia*, and *p_Bacteroidetes* were relatively abundant, and statistically significant differences were observed (*p* < 0.05). The relative abundances of *p_Firmicutes*, *o_Clostridiales*, and *c_Clostridia* were statistically different, and they all belonged to Firmicutes. The relative abundance of *p_Bacteroidetes* was statistically different and belonged to Bacteroidetes. The relative abundance of Firmicutes and Bacteroidetes in the gut microbiota of the PPD-type ginsenoside group was significantly different, which was consistent with the analysis of microbial diversity.

### 3.6. Functional Analysis of Gut Microbiota

**Figure 8:**
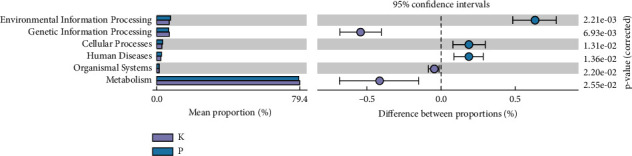
Differential analysis of KEGG metabolic pathways between groups.

KEGG pathway analysis is usually used to analyse the differences and changes in functional genes in the metabolic pathways between samples and groups. As shown in Figure 8, significant differences were observed in seven metabolic categories of the intestinal flora in group P compared with group K. Among them, environmental information processing, cellular processes, and human diseases differed. In conclusion, the intestinal flora of the original ginsenoside group may be involved in this process. However, the correlation between the human intestinal flora and the microbes that changed in the intestinal flora after ginsenoside intervention and the KEGG metabolic pathway remains to be further studied.

## 4. Conclusions

RRLC-Q-TOF MS detection technology was used for the transformation of saponin constituents during sample identification. The human intestinal flora has a metabolic effect on the original ginsenoside; under the action of the intestinal flora in the human gut, the ginsenoside is mainly carried out via deglycosylation, and the ginsenoside is gradually metabolised into secondary aglycones and absorbed into the blood. During biotransformation, the metabolic form and time of the original ginsenosides were relatively stable. PPD-type ginsenosides and their metabolites were present when the sample was transformed to 6. When the sample was transformed for 60 h, most of it existed in the form of Compound K and ginsenoside Rh2, and a few were in the form of ginsenoside PPD. The structure of intestinal flora is complex, and the composition of intestinal flora varies from individual to individual. Bacteria are the main component of intestinal microorganisms, most of which belong to Firmicutes, Bacteroidetes, and Proteobacteria, among which Firmicutes is the dominant phylum. At the phylum level, the P group conspicuously changed the microbial composition in the model group with more Firmicutes and Proteobacteria but fewer Bacteroidetes. At the genus level, compared with group K, the relative abundance of *Escherichia-Shigella* in group P increased, whereas that of Dorea, Prevotella_ 9, and Megasphaera decreased. The proginsenediol saponin group increased the species composition of beneficial bacteria and decreased the relative abundance of harmful bacteria in the intestinal flora at the phylum and genus levels, and the improvement of the intestinal flora may play an important role in disease treatment and health care functions.

## Figures and Tables

**Table 1:** The chemical profile of PPD from *P. ginseng*.

Compound	R1	R2
Rb1	-Glc^2^-^1^Glc	-Glc^6^-^1^Glc
Rc	-Glc^2^-^1^Glc	-Glc^6^-^1^Ara(f)
Rb2	-Glc^2^-^1^Glc	-Glc^6^-^1^Ara(p)
Rb3	-Glc^2^-^1^Glc	-Glc^6^-^1^Xyl
Rd	-Glc^2^-^1^Glc	-Glc
CO	-Glc	-Glc^6^-^1^Ara(p)
Gyp-IX	-Glc	-Glc^6^-^1^Xyl
Gyp-XVII	-Glc	-Glc^6^-^1^Glc
C-Mc1	-Glc	-Glc^6^-^1^Ara(f)
F2	-Glc	-Glc
Rg3	-Glc^2^-^1^Glc	-H
CK	-H	-Glc
Rh2	-Glc	-H
PPD	-H	-H

**Table 2:** RRLC/Q-TOF MS data in the negative ion mode.

No.	*R*_*t*_/min	Compound	Molecular formula	Calculated (m/z)	Measured (m/z)	ESI–MS^2^(-)
M1	20.217	Rb1	C_54_H_92_O_23_	1107.5974	1107.5982	MS^2^[1107.5982]945.5379, 783.4942, 621.4379, 459.3815
M2	20.924	Rc	C_53_H_90_O_22_	1077.5801	1077.5813	MS^2^[1077.5813]945.5409, 783.4933, 621.4366, 459.3841
M3	21.692	Rb2	C_53_H_90_O_22_	1077.5801	1077.5859	MS^2^[1077.5859]945.5419, 783.4936, 621.4388, 459.3839
M4	22.473	Rb3	C_53_H_90_O_22_	1077.5801	1077.5878	MS^2^[1077.5878]945.5418, 783.4937, 621.4362, 459.3883
M5	25.002	Rd	C_48_H_82_O_18_	945.5428	945.5381	MS^2^[945.5381]783.4949, 621.4376, 459.3851
M6	27.052	CO	C_47_H_80_O_17_	915.5323	915.5273	MS^2^[915.5273]783.4809, 621.4381, 459.4814
M7	27.991	Gyp-IX	C_47_H_80_O_17_	915.5323	915.5276	MS^2^[915.5276]783.4813, 621.4367, 459.4851
M8	28.557	Gyp-XVII	C_48_H_82_O_18_	945.5323	945.5380	MS^2^[945.5380]783.4829, 621.4366, 459.4832
M9	29.104	C-Mc1	C_47_H_80_O_17_	915.5323	915.5278	MS^2^[915.5278]783.4803, 621.4399, 459.4813
M10	31.097	F2	C_42_H_72_O_13_	783.4900	783.4849	MS^2^[783.4849]621.4342, 459.3833
M11	32.864	Rg3	C_42_H_72_O_13_	783.4900	783.4851	MS^2^[783.485]621.4338, 459.3842
M12	35.432	CK	C_36_H_62_O_8_	621.4372	621.4483	MS^2^[621.4483]459.3836
M13	35.991	Rh2	C_36_H_62_O_8_	621.4372	621.4449	MS^2^[621.4449]459.3832
M14	40.197	PPD	C_30_H_52_O_3_	459.3893	459.3885	MS^2^[459.3885]221.0667

## Data Availability

The data that support the findings of this study are available from the corresponding author, H. Y., upon reasonable request.

## Supplementary Materials

Supplementary Materials Figure 1. Graphical abstract. In vitro transformation of the proginsenediol saponin group in the human intestinal flora and its effect on the intestinal flora. Figure 2. MS^2^ spectrum of the metabolites of CK, Rh2, and PPD.
