# Supplementary figures and images for: In Vitro Transformation of Protopanaxadiol Saponins in Human Intestinal Flora and Its Effect on Intestinal Flora
Source: Evid Based Complement Alternat Med. 2021 Oct 22;2021:1735803. doi: 10.1155/2021/1735803 (PMC8556111; doi:10.1155/2021/1735803)

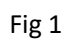

Fig 1

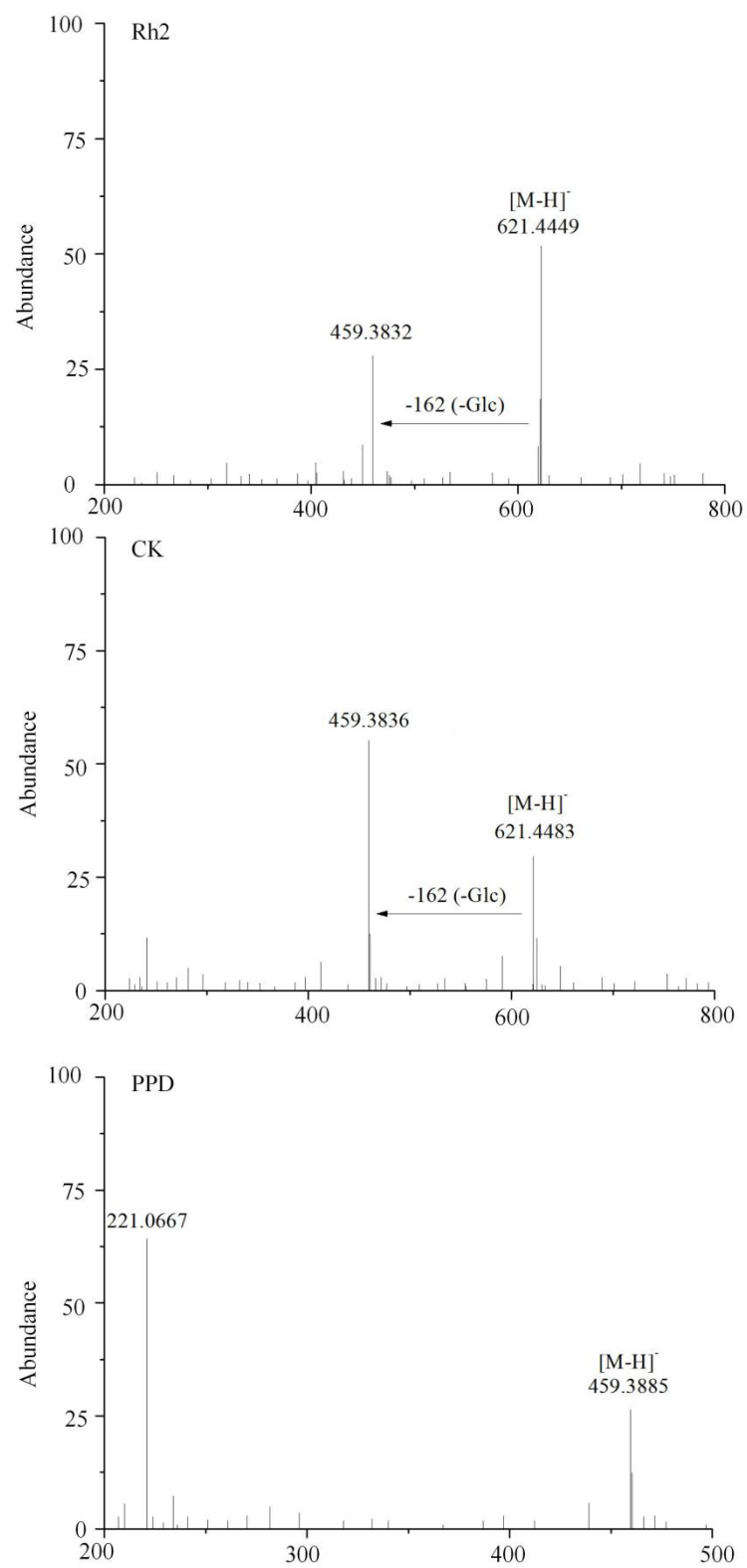

Fig 2

Supplement: Supplementary Materials — Figure 1. Graphical abstract. In vitro transformation of the proginsenediol saponin group in the human intestinal flora and its effect on the intestinal flora. Figure 2. MS2 spectrum of the metabolites of CK, Rh2, and PPD. [file 1735803.f1.pdf]
